# Tofacitinib in Patients Hospitalized With Moderate and Severe COVID-19: Not Just Another Kinase Inhibitor

**DOI:** 10.7759/cureus.52725

**Published:** 2024-01-22

**Authors:** Tharanath Shankar, Akshay Rao, Devisree S, Tejaswini S Hegde, Soumya Sundaresh, Tanvi Sahni, Sushma M Nagaraj

**Affiliations:** 1 Department of Medicine, M. S. Ramaiah Medical College, Bengaluru, IND; 2 Department of Radiology, M. S. Ramaiah Medical College, Bengaluru, IND

**Keywords:** cytokines, corticosteroids, janus kinase inhibitors, covid-19, tofacitinib

## Abstract

Background

There has been an intense search for pharmacological agents that can complement corticosteroid therapy in the treatment of severe coronavirus disease 2019 (COVID-19). The Janus kinase inhibitor tofacitinib has shown promise in this regard. This study aimed to determine the impact of adding tofacitinib to standard care on the mortality and total duration of hospital stay in severe COVID-19.

Methodology

This retrospective study compared the mortality and total duration of hospital stay among patients admitted with severe COVID-19 to a designated COVID-19 hospital in south India who had received tofacitinib in addition to standard care versus standard care alone. Medical case records of severe COVID-19 patients were retrieved and screened for inclusion. Categorical variables such as mortality were expressed as proportions and compared using the chi-square test, while continuous variables such as total duration of hospital stay were compared via the independent t-test. The odds ratio (OR) was calculated for the mortality difference between the two groups. P-values ≤0.05 were considered significant.

Results

Following the initial screening of 250 medical records, 186 patients were included in the final analysis, of whom 103 had received tofacitinib and 83 had received standard care. There was no significant difference in mortality between the two groups (OR = 1.58 (95% confidence interval = 0.71 to 3.51); p = 0.26). The total duration of hospital stay was significantly longer among those in the tofacitinib group (17.14 ± 8.85 days vs. 14.04 ± 5.48 days; p = 0.01).

Conclusions

Tofacitinib did not improve the clinical outcomes when used to supplement corticosteroids in the treatment of severe COVID-19.

## Introduction

The coronavirus disease 2019 (COVID-19) pandemic continues to plague the world with 627,104,342 cumulative cases and 6,567,552 cumulative deaths worldwide to date [1]. Despite a majority of the patients with COVID-19 infection manifesting only mild respiratory symptoms, up to 15% of infections progress to developing hypoxia and respiratory distress [2]. The virus sends the host immune system into overdrive which is thought to be the chief pathogenic mechanism for disease progression. The so-called cytokine storm in severe COVID-19 is mediated by an exponential increase in the levels of inflammatory cytokines such as interleukin-6 (IL-6) and tumor necrosis factor-alpha (TNF-α) [3]. A state of L-phenotype followed by H-phenotype of acute respiratory distress syndrome (ARDS) ensues followed by relentless clinical deterioration [4].

At the beginning of the pandemic, numerous pharmacological agents were investigated as therapeutic options for COVID-19. A heightened understanding of the body’s over-exuberant immune response to the novel virus provided the basis for exploring the role of immunosuppressive drugs in the treatment of severe COVID-19 [5]. The landmark RECOVERY trial helped establish corticosteroids as the standard of care for severe COVID-19. There were dramatic improvements in the treatment outcome of severe COVID-19 after the inclusion of corticosteroids in the treatment regimen. However, during times of surge in COVID-19 cases in the country, even corticosteroids fell short of expectations, and the need for additional agents to curb cytokine storm has been increasingly recognized [6,7]. Although IL-6 antagonists have also shown mortality benefits in studies, they are not widely available and are expensive [7].

Cytokines such as IL-6 and TNF-α exert their effects on immune cells via the signaling cascades of the Janus kinase/signal transduction and activator of transcription (JAK/STAT) pathway. Originally, the acronym JAK stood for just another kinase but the enzyme was subsequently renamed Janus kinase. JAK mediates the phosphorylation of STATs which subsequently translocate into the nucleus to translate for inflammatory mediators. There are four types of JAK enzymes, i.e., JAK1, JAK2, JAK3, and tyrosine kinase 2 (TYK2) [8]. Severe acute respiratory syndrome coronavirus 2 infection triggers inflammation chiefly via the JAK/STAT pathway which leads to the recruitment of pneumocytes, endothelial cells, macrophages, monocytes, lymphocytes, natural killer cells, and dendritic cells that eventually snowball into a cytokine storm. The JAK/STAT signaling also mediates immune responses via B-cell and T-cell differentiation [3]. The JAK/STAT signaling pathway would appear to be a novel target for immunomodulation. JAK inhibitors (JAKi) have been widely used in rheumatology owing to their ability to inhibit the underlying inflammation through this unique mechanism. Another reason why JAKi may be superior to other immunomodulators in COVID-19 therapy is that in addition to their anti-inflammatory effects, they purportedly disrupt cellular endocytosis of the COVID-19 virus [9]. In May 2022, the JAKi baricitinib received Food and Drug Administration approval for the treatment of hospitalized COVID-19 patients who require supplemental oxygen, non-invasive or invasive mechanical ventilation, or extracorporeal membrane oxygenation [10]. Multiple meta-analyses have concluded that there is strong evidence to support the use of baricitinib in the treatment of COVID-19 [10,11].

Tofacitinib is also a widely studied JAKi and is used commonly in rheumatological disorders. It is an orally administered selective inhibitor of JAK1 and JAK3, with functional selectivity for JAK2. In some initial studies that evaluated the role of JAKi in COVID-19, tofacitinib appeared to emerge as a safe and potent anti-COVID-19 agent [12-14]. The findings from one such study even suggested that tofacitinib provided mortality benefits when used in patients with COVID-19 [7]. Yet, a recent meta-analysis found that the evidence for the benefit of tofacitinib use was not as vigorous as that for baricitinib in patients hospitalized with COVID-19 [15]. In early 2022, an advisory from the World Health Organization also advised against the use of tofacitinib in COVID-19 owing to low certainty of evidence [16]. Only a handful of studies have been conducted to assess the role of tofacitinib in COVID-19 therapy. Further research is warranted to explore the effects of tofacitinib in the treatment of severe COVID-19.

In the summer of 2021, India witnessed an unprecedented surge in the number of COVID-19 infections, putting the entire healthcare infrastructure under tremendous strain. The COVID-19 immunization program was still at a nascent stage and its coverage had been limited up to that point. The COVID-19 immunization drive in India was launched on January 16, 2021, with the first phase targeting healthcare and frontline workers. The second phase began on March 1, 2021, and included individuals aged 60 years and above and 45-59 years with comorbidities. The third phase began around April 1, 2021, and targeted individuals aged 45 years and above, and the final phase began by May 1, 2021, targeting individuals aged above 18 years and above [17]. Healthcare facilities were overburdened with patients suffering from severe COVID-19. There developed a crunch for hospital beds, medications, and even oxygen supplies [18,19]. Despite treatment with corticosteroids, anticoagulants, and respiratory support, the absolute number of casualties being observed was tragically high. In times of such despair, health authorities began to advocate for the incorporation of additional immunomodulatory agents in the COVID-19 treatment regimen. An example of this was the recommendation to use tofacitinib to supplement corticosteroids in the treatment of patients with severe COVID-19 [20]. This off-label tofacitinib add-on therapy was offered to several patients with severe disease at our designated COVID-19 hospital during the summer of 2021. Hence, we sought to retrospectively analyze the impact of tofacitinib on the treatment outcomes of patients with severe COVID-19 at our center.

## Materials and methods

Study design

This retrospective case-control study included patients with moderate and severe COVID-19 who had been admitted to a designated COVID-19 hospital in south India. The patients were assigned to either the control group that received standard care or the study group that received tofacitinib in addition to standard care. Institutional review board approval was obtained for the study (approval number: XXXMC/EC/SP-04/11-2021). Medical records of 250 patients were screened, of whom 180 were included in the study. The inclusion and exclusion criteria are presented in Table 1.

**Table 1:** Inclusion and exclusion criteria. COVID-19 = coronavirus disease 2019; SARS-CoV-2 = severe acute respiratory syndrome coronavirus 2

Inclusion criteria	Exclusion criteria
Patients aged 18 years and above who were hospitalized for SARS-CoV-2 infection, diagnosed by real-time polymerase chain reaction testing	Patients who had been receiving tofacitinib or any other immunosuppressive medications before the diagnosis of COVID-19 infection
Patients with peripheral oxygen saturation levels below 94%	Patients with pre-existing hepatic dysfunction, anemia, leucopenia or thrombocytopenia, or chronic kidney disease stage IIIb or higher (estimated glomerular filtration rate <45 mL/minute/1.73m^2^)
Patients who at admission were initiated on standard care for severe COVID-19	Past or current thrombotic events
	Death within 48 hours of hospitalization
	If any alternate immunomodulator drugs apart from tofacitinib were used to supplement corticosteroid therapy (including tocilizumab)
	Patients who had received one or more doses of COVID-19 vaccines

Treatment

Standard care for moderate and severe COVID-19 included corticosteroid therapy (parenteral methylprednisolone at 40 mg/day); anticoagulation in the form of low-molecular-weight heparin at 1 mg/kg twice daily; oxygen support via a non-rebreathing mask, high-flow nasal oxygen therapy, and non-invasive and invasive mechanical ventilation; prophylactic antibiotic coverage with ceftriaxone; and remdesivir at the dose of 200 mg on the first day, followed by 100 mg once a day for four days intravenously.

During the peak of the outbreak in May 2021, the designated COVID-19 hospital had framed an institutional policy regarding the emergency use of tofacitinib. Tofacitinib therapy was offered to all patients with severe COVID-19 who had not shown satisfactory response to standard care for 48 hours since admission (five days after the day of COVID-19 symptom onset and requiring oxygen supplementation greater than 5 L per minute via a face mask despite being on optimal steroid therapy). Detailed discussions were held with the patient’s relatives, and, whenever possible, with the patients before initiating tofacotinib. The nature of the disease, the lack of improvement despite instituting standard care, and the potential risks and benefits of administering tofacitinib were discussed before obtaining consent to start tofacitinib add-on therapy. Before initiating tofacitinib, the following conditions were confirmed: complete blood counts, liver function tests, and serum procalcitonin levels were normal; C-reactive protein was greater than 5 mg/dL; and viral serology tests for human immunodeficiency virus and hepatitis B and C were non-reactive.

Tofacitinib was administered orally at a dose of 10 mg twice daily for the first five days followed by 5 mg once daily for the next five days and then stopped. The complete hemogram and liver function tests were monitored every 48 hours for all patients on tofacitinib. In the event of abnormalities in these tests, tofacitinib was discontinued.

Outcome measures

The primary outcome measured was mortality in both groups. The total duration of hospital stay was also compared between the two groups. The complications and adverse effects developing during their hospital stay were also documented.

Sample size

In the study by Maslennikov et al., it was observed that the mortality among the group of patients with severe COVID-19 who had received standard care with and without tofacitinib was 16.6% and 40%, respectively [12]. In the present study expecting a similar result with 80% power, 95% confidence interval (CI), and an estimated risk difference of 23.4%, it would require a minimum of 40 subjects to be included in each group.

Data extraction

Medical case records of patients hospitalized at the designated COVID-19 hospital were accessed. The clinical information, demographic details, history of comorbidities, and COVID-19 disease severity were extracted from the records of patients who were eligible for inclusion in the study. Based on the treatment received, they were divided into the standard care vs. the tofacitinib group. Their laboratory investigation profiles were screened and relevant values of their reports such as complete hemogram, liver function tests, serum creatinine, and electrolytes were documented. Patients underwent thoracic computed tomography (CT) if their condition permitted it. Parameters assessed included the presence of ground-glass opacity, consolidation, nodule, reticulation, interlobular septal thickening, crazy-paving pattern, linear opacities, etc. The chest CT scoring system was used to grade the severity of COVID-19. Based on the extent of lung parenchymal involvement, the chest CT score was calculated for each of the five lobes (Table 2).

**Table 2:** Chest CT scoring. Li et al. (2020) [21]. CT = computed tomography

Score	Percentage of lung parenchymal involvement
0	No involvement
1	<5
2	5–25
3	26–50
4	51–75
5	>75

The total score which was the sum of the scores of all five lobes ranging from 0 (no parenchymal involvement) to 25 (>75% involvement of all five lobes) was calculated [21].

The total duration of hospital stay was calculated after excluding patients who had died. Among patients in the control group, each of their duration of stay was calculated from the date of admission to the date of their discharge from the hospital. For the patients in the tofacitinib group, the duration of stay was calculated from the date of tofacitinib initiation to the date of their discharge from the hospital. This was because tofacitinib had been added only after 48 hours of failure to respond to standard care. Finally, the number of patients who died in each group, as well as complications and adverse effects were noted.

Statistical analysis

Descriptive statistics of mortality were analyzed and summarized as percentages. The association between tofacitinib and mortality was expressed as odds ratios with 95% CIs. The chi-square test was used to determine the statistical significance of the comparison of mortality between the two groups. Other descriptive variables were analyzed similarly. The total duration of hospital stay was summarized in terms of means with standard deviations. The independent t-test was used to compare the total duration of hospital stay between the two groups, as well as for other continuous variables. A p-value ≤0.05 was considered significant.

## Results

**Figure 1:**
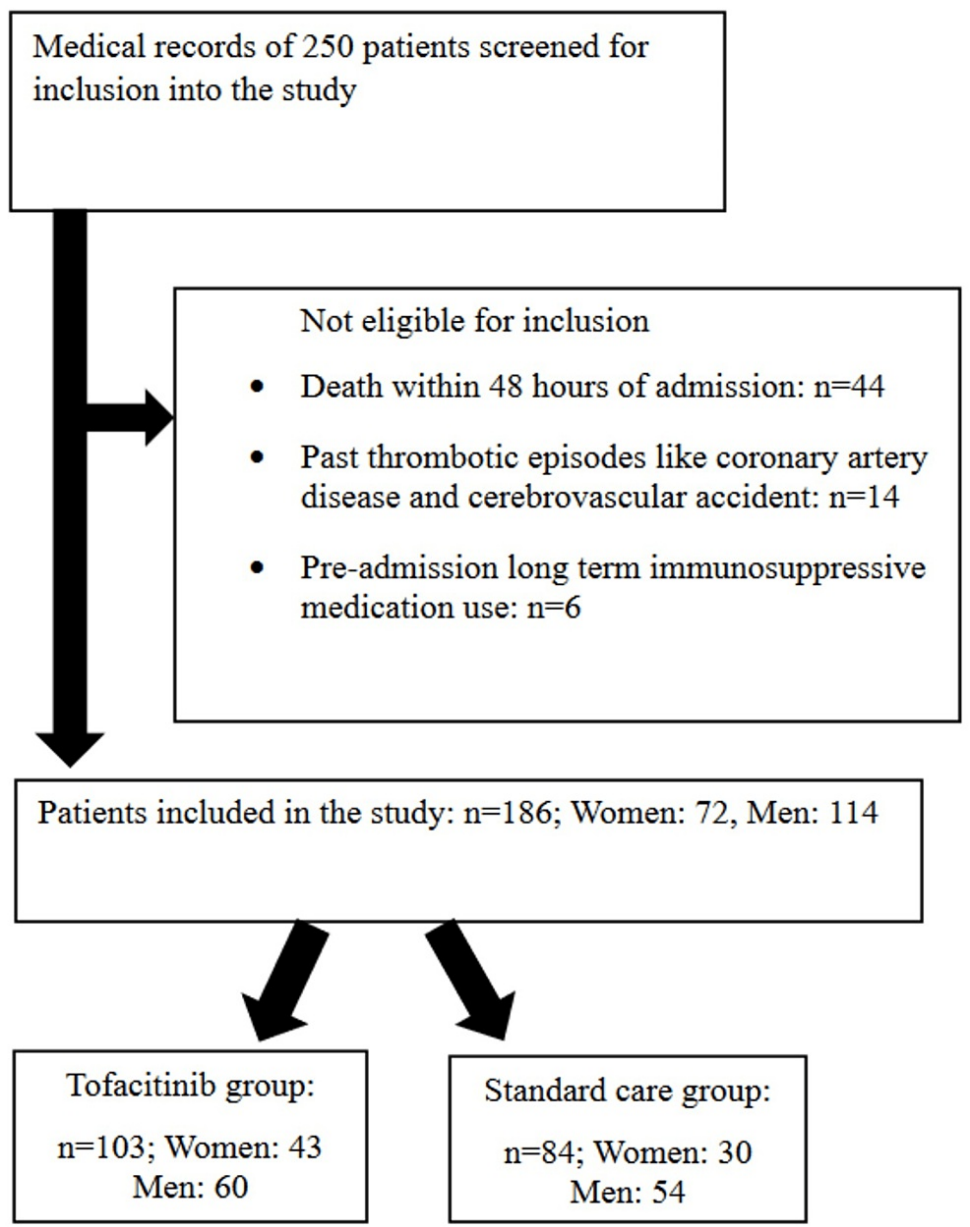
STROBE flow diagram.

Overall 250 medical records were screened for inclusion in the study. After the exclusion of 64 patients, a total of 186 patients were included and their medical records were analyzed. Among them, 103 had received tofacitinib, and the remaining 83 had received standard care. The STROBE flow diagram in Figure 1 summarizes the same.

The baseline characteristics of the two groups are summarized in Table 3. Notably, the patients in the tofacitinib group were significantly younger. There were no significant differences between the two groups concerning gender distribution, category of COVID-19, or comorbid conditions.

**Table 3:** Baseline characteristics of patients in the two groups. ^#^: CT thorax could be performed only for 57 patients in the tofacitinib group and 38 patients in the standard care group. COVID-19 = coronavirus disease 2019; CT = computed tomography

Parameter		Tofacitinib group (n = 103)	Standard care group (n = 83)	P-value
Age (year)		52.51 ± 13.82	59.74 ± 15.72	0.003
Male gender (%)		60	54	0.34
COVID-19 severity	Moderate	35	31	0.57
	Severe	68	52	
Hypertension (%)		25 (33.73)	28 (24.27)	0.15
Diabetes mellitus (%)		38 (36.89)	41 (49.40)	0.08
CT severity score^#^		16.75 ± 4.21	14.42 ± 4.37	0.28

**Figure 2:**
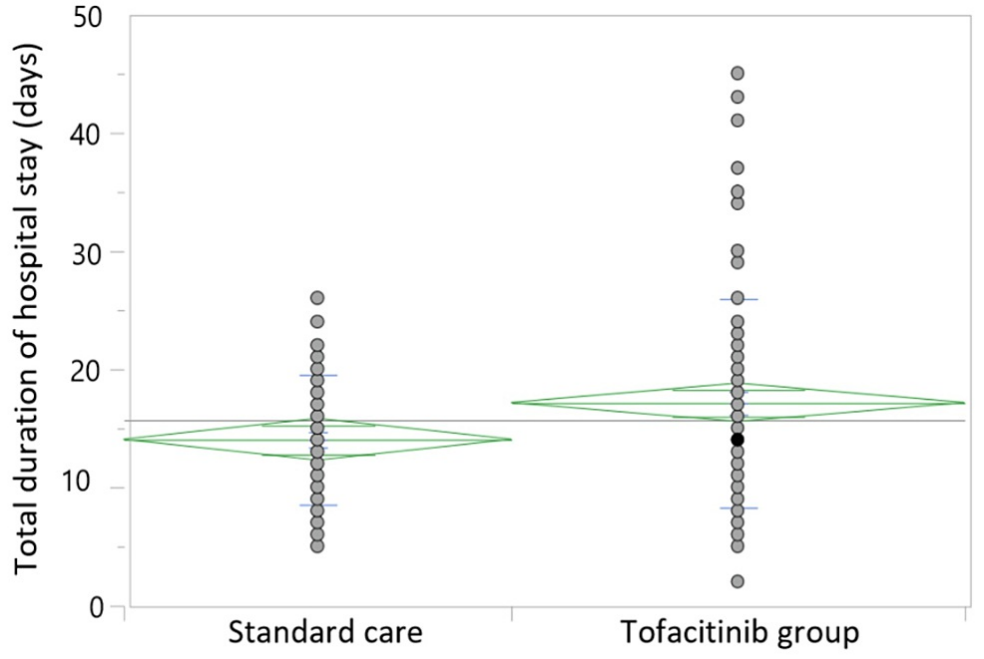
Comparison of the total duration of hospital stay.

The comparison of the findings regarding the total duration of hospital stay, number of deaths, and complications between the two groups is summarized in Table 4. The total duration of hospital stay was significantly longer among those in the tofacitinib group (17.14 ± 8.85 days vs. 14.04 ± 5.48 days; p = 0.01), as depicted in Figure 2. There was no significant difference in mortality between the two groups (OR = 1.58 (95% CI = 0.71 to 3.51); p = 0.26). The rates of complications in the two groups also did not differ significantly.

**Table 4:** Comparison between the two groups regarding the total duration of hospital stay, deaths, and complications observed. OR = odds ratio; CI = confidence interval

	Tofacitinib group (n = 103)	Standard care group (n = 83)	P-value
Total duration of hospital stay	17.14 (±8.85)	14.04 (±5. 48)	0.01
Total deaths	20	11	0.26 (OR = 1.58; 95% CI = 0.71-3.51)
Secondary infections	6	5	0.95
Acute kidney injury	2	3	0.40
Dyselectrolytemia	7	2	0.16
Leucopenia/Thrombocytopenia	1	2	0.43
Elevation in transaminases	6	4	0.76

## Discussion

In this study, the use of tofacitinib did not appear to improve the outcomes in severe COVID-19 compared to standard care. Tofacitinib did not reduce the mortality in comparison to standard care for severe COVID-19. Previous studies on tofacitinib use in COVID-19 have yielded conflicting results. A Russian study conducted early in the pandemic claimed that five days of tofacitinib therapy significantly improved the survival rates (84.4% vs. 60.0%; p = 0.009) in COVID-19 patients. Although it was a retrospective study that included only 62 patients, the investigators made extensive efforts to control for multiple confounding factors [12]. In 2020, a major pharmaceutical company funded a placebo-based, multicenter, double-blinded randomized controlled trial (RCT) in Brazil to assess the role of tofacitinib therapy given for two weeks in COVID-19 pneumonia [20]. Although the study was not powered to analyze the difference in mortality outcomes alone, the total number of deaths on day 28 among those participants who received tofacitinib (4/144) against those who received standard care (8/145) did not appear to differ significantly (hazard ratio = 0.49; 95% CI = 0.15-1.63). Thereafter the same pharmaceutical company also funded an RCT in India, in which 100 patients infected with COVID-19 had been enrolled, 50 of whom eventually received tofacitinib. This RCT too failed to demonstrate any mortality benefit with the use of tofacitinib. No deaths were observed in this RCT, possibly due to the inclusion of only patients with mild-to-moderate disease [13]. Another small retrospective study from India found no significant difference in COVID-19 mortality rates with the use of tofacitinib against standard care [22].

Counterintuitively, the total duration of hospital stay in this study was seen to be significantly longer among the survivors of severe COVID-19 in the tofacitinib group. The reason behind this unexpected finding is unclear. Although immunosuppression may result in secondary bacterial infections/opportunistic infections, thereby prolonging hospital stay, there was no significant difference between the two groups regarding this. Delayed recovery of the lung from the underlying COVID-19 infection seems to be the most likely explanation for their extended hospital stay. Some previous authors have reported that tofacitinib does not significantly impact the duration of hospital stay [13]. A meta-analysis from the Cochrane database also reported with moderate certainty of evidence, that JAKi probably leads to little or no improvement in the clinical status of patients with severe COVID-19 [10].

In this study, adverse events associated with tofacitinib were not significantly higher than in those who received standard care. Despite the low incidence of adverse events with the use of tofacitinib, the findings of this study dissuade its use in the treatment of COVID-19 due to the lack of clinical benefits. On the contrary, there appears to be more robust evidence favoring the use of baricitinib in the treatment of severe COVID-19 [9,10]. Baricitinib is a selective JAK1 and JAK2 antagonist whereas tofacitinib chiefly inhibits JAK1 and JAK3 with only modest inhibition of JAK2. Moreover, unlike tofacitinib, some studies have proposed that baricitinib may possess direct antiviral properties [9,10]. These differences may explain the superiority of baricitinib over tofacitinib in improving the outcomes when used in severe COVID-19.

Limitations of this study include its retrospective study design and the limited sample size. The data retrieved was from a period when healthcare resources were being stretched to unprecedented levels. As details regarding the mode of ventilation were not captured, there is a possibility that sampling bias cannot be excluded. Moreover, the details of thrombotic complications were not captured. Owing to the unavailability of hospital beds, patients would be hospitalized in the advanced stages of their illness. In these extraordinary circumstances, patients who would otherwise warrant intensive care were managed in general ward settings. Yet, with the inclusion of only non-immunized patients in this study, it hopes to offer an unadulterated insight into the effects of tofacitinib in severe COVID-19. Larger prospective randomized studies may help to confirm these findings.

## Conclusions

Tofacitinib did not improve the clinical outcomes when used to supplement corticosteroids in the treatment of severe COVID-19. Further studies with larger sample sizes and RCT study designs are required for conclusive evidence on the role of tofacitinib in severe COVID-19.
